# The evolution of mitochondrial genomes in modern frogs (Neobatrachia): nonadaptive evolution of mitochondrial genome reorganization

**DOI:** 10.1186/1471-2164-15-691

**Published:** 2014-08-20

**Authors:** Yun Xia, Yuchi Zheng, Ikuo Miura, Pamela BY Wong, Robert W Murphy, Xiaomao Zeng

**Affiliations:** Chengdu Institute of Biology, Chinese Academy of Sciences, Chengdu, 610041 China; Insititute for Amphibian Biology, Graduate School of Science, Hiroshima University, Kagamiyama 1-3-1, Higashi-Hiroshima, 739-8526 Japan; Centre for Biodiversity and Conservation Biology, Royal Ontario Museum, 100 Queen’s Park, Toronto, ON M5S 2C6 Canada

**Keywords:** Mitogenomics, tRNA gene, Gene rearrangement, Gene duplication, Random gene loss, Gene order

## Abstract

**Background:**

Although mitochondrial (mt) gene order is highly conserved among vertebrates, widespread gene rearrangements occur in anurans, especially in neobatrachians. Protein coding genes in the mitogenome experience adaptive or purifying selection, yet the role that selection plays on genomic reorganization remains unclear. We sequence the mitogenomes of three species of *Glandirana* and hot spots of gene rearrangements of 20 frog species to investigate the diversity of mitogenomic reorganization in the Neobatrachia. By combing these data with other mitogenomes in GenBank, we evaluate if selective pressures or functional constraints act on mitogenomic reorganization in the Neobatrachia. We also look for correlations between tRNA positions and codon usage.

**Results:**

Gene organization in *Glandirana* was typical of neobatrachian mitogenomes except for the presence of pseudogene *trnS* (*AGY*). Surveyed ranids largely exhibited gene arrangements typical of neobatrachian mtDNA although some gene rearrangements occurred. The correlation between codon usage and tRNA positions in neobatrachians was weak, and did not increase after identifying recurrent rearrangements as revealed by basal neobatrachians. Codon usage and tRNA positions were not significantly correlated when considering tRNA gene duplications or losses. Change in number of tRNA gene copies, which was driven by genomic reorganization, did not influence codon usage bias. Nucleotide substitution rates and d_N_/d_S_ ratios were higher in neobatrachian mitogenomes than in archaeobatrachians, but the rates of mitogenomic reorganization and mt nucleotide diversity were not significantly correlated.

**Conclusions:**

No evidence suggests that adaptive selection drove the reorganization of neobatrachian mitogenomes. In contrast, protein-coding genes that function in metabolism showed evidence for purifying selection, and some functional constraints appear to act on the organization of rRNA and tRNA genes. As important nonadaptive forces, genetic drift and mutation pressure may drive the fixation and evolution of mitogenomic reorganizations.

**Electronic supplementary material:**

The online version of this article (doi:10.1186/1471-2164-15-691) contains supplementary material, which is available to authorized users.

## Background

Organization of metazoan mitochondrial genomes (mitogenomes) is usually conserved [1]. Notwithstanding, many cases of genome reorganization (e.g., rearrangement, duplication and loss) occur in closely related animals [2–4]. Typically, the mitogenome of metazoans encodes 13 protein-coding genes along with 2 rRNAs and 22 tRNAs. The 13 protein-coding genes, all of which play vital roles in the respiration chain, producing 95% of the adenosine triphosphate (ATP) required for cellular energy through oxidative phosphorylation [5]. The function of these proteins results in the distribution of mitochondrial DNA (mtDNA) diversity being far from random [6]. Evidence for adaptive evolution of mtDNA genes exists in some vertebrate lineages, such as mammals and reptiles [7–9].

Many studies have indicated that protein-coding genes of the mitogenome experienced adaptive or purifying selection, yet the role that selective pressure plays on reorganization of the mitogenome is subject to debate. On the one hand, mitogenomic organization may evolve neutrally [10]. Dowton et al. [2] characterized 67 gene arrangements in the Hymenoptera and suggested that tRNAs occupy selectively neutral positions. Furthermore, Boussau et al. [11] proposed that mitogenomic structural evolution (i.e., gene duplication) was influenced by population size. The genomic duplication is more likely to occur in lineages where the efficiency of selection had been reduced and the ratio of nonsynonymous to synonymous substitution (d_N_/d_S_) increased. These findings indicate that mitogenomic reorganization accompanies lower or relaxed selection, and that fixation of the structural alteration is nonadaptive [11, 12]. On the other hand, positive selection could also act on gene order in the mitogenome. The location of highly transcribed RNAs (such as 12S and 16S rRNA) is adjacent to transcriptional regulatory elements in the control region (CR) [13]. Significant correlations between codon usage and tRNA positions in vertebrate mt genomes (e.g., [14]), suggest that frequently transcribed tRNA genes, such as hydrophobic residues, also occur close to the CR due to functional efficiency. Loss of a duplicated gene may not occur randomly, but rather retention may depend on the distribution of the copies [15]. Thus, changes in tRNA gene positions could owe to adaptive selection [14].

Variation in number of tRNA genes may influence codon usage bias in mitogenomes. Genome reorganization of mtDNA has been linked to variation in the number of tRNA genes [16]. Translational selection may drive the coevolution of tRNA genes and codon usage [17]. Positive correlations between tRNA abundance and codon usage bias have been observed in some unicellular (e.g., *Saccharomyces cerevisiae*) and multicellular (e.g., *Caenorhabditis elegans*) eukaryotic genomes [18, 19]. In mitochondria, oxidative phosphorylation often requires a few cytosolic tRNAs encoded by nuclear DNA [20], and these imported tRNAs could compensate for changes in the number of mt tRNA genes. Consequently, the influence asserted by changes in the number of tRNA genes and the role played by selection on mitogenome rearrangements remains elusive [21]. An understanding of selective constraints on mitogenomic reorganization might provide some clarity on the mechanisms that underlie mitogenome evolution. Unfortunately, little is known about how gene duplications, losses, and rearrangements of tRNA genes influence codon usage of mt genes due to the paucity of examples of mitogenomic reorganization within closely related species. Among vertebrates, anurans (especially neobatrachians) facilitate revealing the relationship between tRNA genes positions and codon usage due to the high levels of gene rearrangements [22–24]. The variation also allows for explorations into how variation in tRNA gene copy number influences codon usage bias.

Most mitogenomes of non-neobatrachians (e.g., Archaeobatrachia) have the vertebrate ancestral gene order (AGO) [25, 26]; *Leiopelma archeyi* and *Leptolalax pelodytoides* are exceptions [24, 27]. In contrast, rearranged gene orders (RGO) characterize neobatrachians [28, 29], which share *LTPF* clusters (*trnL*-*trnT*-*trnP*-*trnF*) resulted from rearrangements of four typical vertebrate tRNA genes [29–31]. Further, recurrent gene rearrangements involve duplications and/or losses of CR and tRNA genes [3, 32–35]. These findings contrast with the proposition that vertebrates possess highly conserved mitogenomic organizations [1, 36].

Accelerated mt substitution rates in protein-coding genes occur in neobatrachians. Such could be a consequence of relaxed purifying selection in the ancestor of neobatrachians [23, 37]. However, highly rearranged mt genomes and high rates of nucleotide substitution are not significantly correlated [23, 37]. Studies on the correlation between tRNA gene rearrangements and codon usage could provide new insights into the role played by selection on mitogenomic reorganization.

Herein, we sequence the mt genome of three congeneric species of *Glandirana* and three hotspots of gene rearrangements across 20 species in the Ranoides. We supplement these data with sequences from GenBank to examine the relationship between tRNA gene arrangements and codon usage in anurans. Our results suggest selection does not favor any specific rearrangement of gene position. Further, tRNA gene duplications or losses do not appear to influence codon usage bias. Our findings shed light on the non-adaptive evolution of mitogenomic reorganization, at least in neobatrachians.

## Results and discussion

### *trnS*(*AGY*) pseudogene of *Glandirana*and gene rearrangement in Ranidae

We failed to sequence the complete mt genome of *Glandirana rugosa*, *G. emeljanovi*, and *G. tientaiensis* (Table 1, [GenBank: KF771341–KF771343]) due to long, highly repetitive nucleotides in the CRs. All three mtDNA genomes contained 13 protein-coding genes, two rRNA genes, 21 tRNA genes [Ψ *trnS* (*AGY*)], and non-coding regions (Additional file 1). The mt genomes of *Glandirana* were arranged identically to those typical of other neobatrachians, except for the presence of *trnS* (*AGY*) pseudogenes (Additional files 1, 2). Coding genes were similar in length to their counterparts in other anurans. Differences in mtDNA genome size and organization of *Glandirana* owed to the size of repeat-sequences in the CR (Additional file 1).

**Table 1:** **Data for samples of employed Ranidae and mt regions sequenced in this study**

Species	Voucher number	Collection locality	Sequenced region (GenBank Accession number)			
			12S–16S	*nad2*–*cox1*	*nad3–nad5*	*nad5–cob*
*Glandirana rugosa*	CIB IM3	Hiroshima, Japan	Partial mitochondrial genome			
			(KF771341)			
*Glandirana emeljanovi*	XM3124	Huanren, Liaoning, China	Partial mitochondrial genome			
			(KF771343)			
*Glandirana tientaiensis*	QLY277	Ninghai, Zhejiang, China	Partial mitochondrial genome			
			(KF771342)			
*Amolops chunganensis*	QLY313	Shenlongjia, Hubei, China	12S–V–16S	WA'N'O_L_ANO_L_'CY	nad3–R–nad4L–nad4–H–S–nad5	
			(KF771285)	(KF771328)	(KF771305)	
*Amolops granulosus*	QLY311	Shenlongjia, Hubei, China	12S–V–16S	WA'N'O_L_ANO_L_'CY	nad3–R–nad4L–nad4–H–S–nad5	
			(KF771286)	(KF771329)	(KF771306)	
*Amolops kangtingensis*	XM999	Kangding, Sichuan, China	12S–V–16S	WA'N'O_L_ANO_L_'CY	nad3–R–nad4L–nad4–H–S–nad5	
			(KF771287)	(KF771330)	(KF771307)	
*Amolops loloensis*	XM031	Hongya, Sichuan, China	12S–V–16S	WA'N'O_L_ANO_L_'CY	nad3–R–nad4L–nad4–H–S–nad5	
			(KF771288)	(KF771331)	(KF771308)	
*Amolops mantzorum*	XM3127	Dayi, Sichuan, China	12S–V–16S	WA'N'O_L_ANO_L_'CY	nad3–R–nad4L–nad4–H–S–nad5	
			(KF771289)	(KF771332)	(KF771309)	
*Amolops ricketti*	XY21	Leishan, Guizhou, China	12S–V–16S	WANO_L_CY	nad3–R–nad4L–nad4–H–S–nad5	
			(KF771290)	(KF771333)	(KF771310)	
*Amolops wuyiensis*	QLY53	Qingyang, Anhui, China	12S–V–16S	WANO_L_CY	nad3–R–nad4L–nad4–H–S–nad5	
			(KF771291)	(KF771334)	(KF771311)	
*Babina adenopleura*	XM2827	Wuyi, Fujian, China	12S–V–16S	WANO_L_CY	nad3–R–nad4L–nad4–H–S–nad5	nad5–noncoding–nad6–E–cob
			(KF771281)	(KF771324)	(KF771301)	(KF771319)
*Babina pleuraden*	XM2958	Lijiang, Yunnan, China	12S–V–16S	WANO_L_CY	nad3–R–nad4L–nad4–H'–S–nad5	
			(KF771283)	(KF771326)	(KF771303)	
*Hylarana latouchii*	XM2852	Xiangshan, Zhejiang, China	12S–V–16S	WANO_L_C'Y	nad3–R–nad4L–nad4–H–S–nad5	nad5–nad6–E–cob
			(KF771284)	(KF771327)	(KF771304)	(KF771321)
*Hylarana nigrovittata*	200905293	Mengla, Yunnan, China	12S–V–16S	WANO_L_C'Y	nad3–R–nad4L–nad4–H–S–nad5	
			(KF771300)	(KF771340)	(KF771318)	
*Rana chaochiaoensis*	CQ004	Jingdong, Yunnan, China	12S–V–16S	WANO_L_CY	nad3–R–nad4L–nad4–H–S–nad5	nad5–noncoding–nad6–E–cob
			(KF771282)	(KF771325)	(KF771302)	(KF771320)
*Rana chensinensis*	XM827	Wanyuan, Sichuan, China	12S–V–16S	WANO_L_CY	nad3–R–nad4L–nad4–H–S–nad5	
			(KF771299)	(KF771339)	(KF771317)	
*Odorrana andersonii*	XM3206	Jingdong, Yunnan, China	12S–V–16S		nad3–R–nad4L–nad4–S–nad5	
			(KF771292)		(KF771312)	
*Odorrana grahami*	3LW0015	Lijiang, Yunnan, China	12S–V–16S		nad3–R–nad4L–nad4–S–nad5	
			(KF771293)		(KF771313)	
*Odorrana livida*	QLY214	Wenzhou, Zhejiang, China	12S–V–16S	WANO_L_CY	nad3–R–nad4L–nad4–S–nad5	nad5–noncoding–nad6–E–cob
			(KF771294)	(KF771335)	(KF771314)	(KF771323)
*Odorrana lungshengensis*	XY50	Leishan, Guizhou, China	12S–V–16S	WANO_L_CY		
			(KF771295)	(KF771336)		
*Odorrana margaratae*	XM3519	Dayi, Sichuan, China	12S–V–16S		nad3–R–nad4L–nad4–S–nad5	
			(KF771296)		(KF771315)	
*Odorrana schmackeri*	QLY80	Qingyang, Anhui, China	12S–V–16S	WA N'O_L_C' NO_L_'CY		nad5–noncoding–nad6–E–cob
			(KF771297)	(KF771337)		(KF771322)
*Odorrana versabilis*	XY86	Leishan, Guizhou, China	12S–V–16S	WANO_L_ N'O_L_' CY	nad3–R–nad4L–nad4–S–nad5	
			(KF771298)	(KF771338)	(KF771316)	

Abbreviations for genes and non-coding regions are from MitoZoa (http://www.caspur.it/mitozoa). “'” denotes pseudogenes.

**Figure 1:**
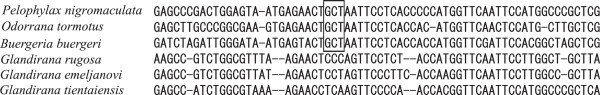
**Aligned sequences for the segment of coding** ***trnS (AGY)*** **of** ***Pelophylax nigromaculata*** **,** ***Odorrana tormota*** **,** ***Buergeria buergeri*** **, and the corresponding segment of** ***Glandirana*** **.** The anticodons of *trnS (AGY)* shown in the frame.

We re-designed a pair of PCR primers that were conserved in *Glandirana* (data not shown) to amplify this region [GenBank: KF771278–KF771280] for confirming the presence of *trnS* (*AGY*) pseudogenes. All three mitogenomes in *Glandirana* included 62–63 bp of a non-coding sequence downstream of *trnH* in the typical location of *trnS* (*AGY*). The primary sequence of *trnS* (*AGY*) in *Glandirana* was very similar to those in the other frogs (Figure 1), though anticodons differed from the typical canonical sequence GCT and among the three species (CCC in *G. rugosa*; CTA in *G. emeljanovi*; and TCA in *G. tientaiensis*). With the exception of the original position of *trnS* (*AGY*), homologous fragments were not found. As previously reported in *G. rugosa* [22], pseudogene *trnS* (*AGY*) occurred in all of our *Glandirana*. This occurrence constituted a synapomorphy for the three species.

**Figure 2:**
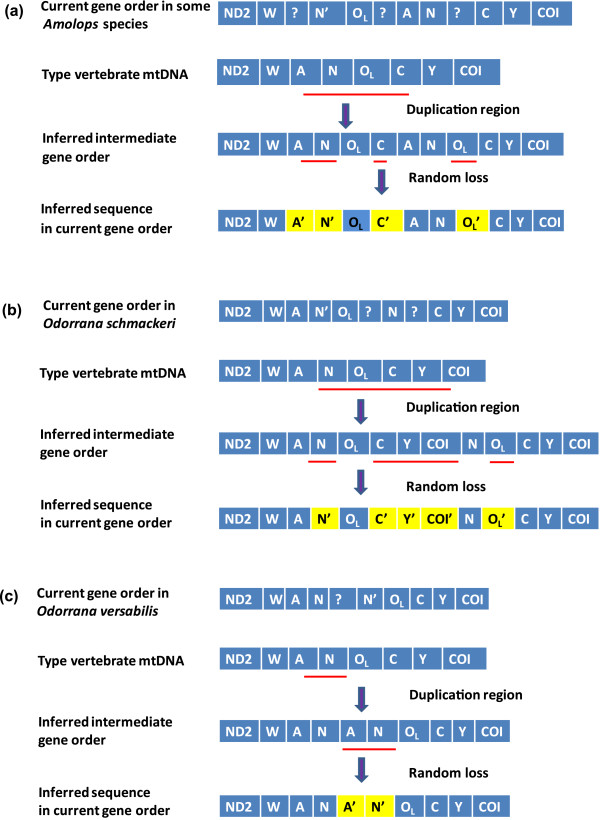
**Putative mechanism of gene rearrangement of the mitochondrial sequences according to a model of tandem duplication of gene regions and subsequent gene deletions.** *WANCY* gene rearrangement in **(a)** *Amolops chunganensis*, *A. granulosus*, *A. kangtingensis*, *A. loloensis*, and *A. mantzorum*, **(b)** *Odorrana schmackeri*, and **(c)** *O. versabilis*.

Our study identified gene rearrangements in three ranid hotspot fragments (Table 1). The typical gene order of the *trnW*–*trnY* block was *trnW*, *trnA*, *trnN*, origin of light strand replication (O_L_), *trnC*, and *trnY* (*WANCY*). In some species of *Amolops* and *Odorrana*, three gene rearrangements differed from the consensus order across vertebrates. The tandem duplication–random loss (TDRL) model provided a plausible mechanism for these rearrangements [38, 39]. The hypothesized duplicated region in the mitogenome of *Amolops chunganensis*, *A. granulosus*, *A. kangtingensis*, *A. loloensis*, and *A. mantzorum* included a partial fragment of *trnA*, all of *trnN* and O_L_, and a partial fragment of *trnC* (Figure 2a). The inferred duplications in the mitogenome of *Odorrana schmackeri* included all of *trnN*, O_L_, *trnC*, *trnY*, and partial *cox1* (~264 bp) (Figure 2b). The hypothesized duplicated region in the mtDNA of *O. versabilis* included a partial fragment of *trnA*, and all of *trnN* (Figure 2c). In the *WANCY* fragments, gene rearrangements were detected for *O. schmackeri*, *O. versabilis* and the five species of *Amolops*, but differences in the duplicated fragments and the position of the pseudogenes or superfluous gene copies suggested these features originated separately (Figure 2). The *WANCY* region was reported to be a hotspot for gene order rearrangement in amphibians [39, 40], and our discovery was consistent with these findings.

All analyzed species of *Odorrana* shared a translocation of *trnH*, which moved to CR from between *nad4* and *trnS (AGY)*. This result was consistent with the previous observations [22, 32]. The original *trnH* in *Babina pleuraden* also become a pseudogenes. Owing to shared patterns, Kakehashi et al. [41] proposed that the *trnH*–*trnE* block was duplicated in the ancestral lineage of *Babina* and *Odorrana*. However, *Babina okinavana*, *B. holsti,* and *B. subaspera* have functional *trnH* genes in the ancestral position [41]. These results suggest that random losses the duplications may drive the differences in gene order in closely-related species.

An inter-genic spacer occurs between *nad5* and *nad6* in *Babina adenopleura* and *Odorrana schmackeri* at lengths of 457 bp and 306 bp, respectively. We cannot identify the noncoding sequences and no evidence suggests they were homologous fragments. This evidence indicates independent origins.

### Genomic features and phylogenetic relationship

**Figure 3:**
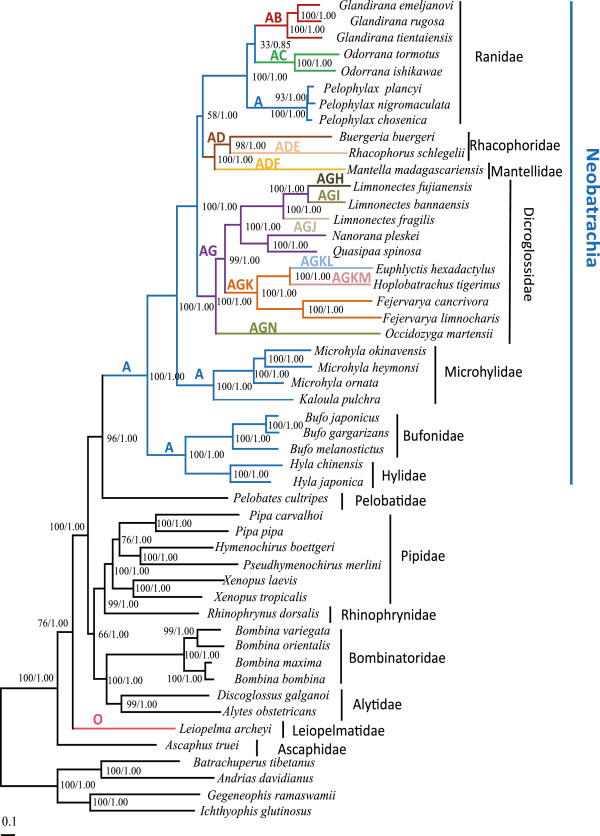
**A maximum likelihood phylogeny of the derived from 13 coding protein of mtDNA sequences for Anura.** Bayesian inference obtained the same topology. Numbers above the lines or beside the nodes were rapid bootstrap proportions calculated with 1000 replicates and Bayesian posterior probabilities, respectively. The branch lengths are to scale. Mt genomic features that are useful markers for inferring relationships, and genomic features of each species are labeled on tree as follows: **(A)** *LTPF* cluster; **(B)** *trnS (AGY)* pseudogene; **(C)** translocation of *trnH*; **(D)** translocation of *nad5*; **(E)** *LTPF* changed into *TLPF* cluster; **(F)** duplication of CR and *trnM*; **(G)** duplication of *trnM*; **(H)** translocation of *trnE* and loss one copy of *trnM*; **(I)** *trnA*, *trnN*, *trnC* and *trnE* gene loss; **(J)** *trnQ*, *trnA*, *trnN*, *trnC*, *trnY*, and *trnP* gene loss; **(K)** translocation of *nad5*; **(L)** translocation of *trnP*; **(M)** translocation of *trnL(CUN)*; **(N)** *WANCY* changed into *WACYN*; **(O)** translocation of *nad5*, *trnE*, and *trnP*.

The final concatenated alignment of our mtDNA dataset for 50 species contained 10836 nucleotide positions, including 7361 variable sites of which 6746 were potentially parsimony-informative. Maximum likelihood (ML) and Bayesian inference (BI) methods of phylogenetic reconstruction obtained in the same tree topologies for 13 mt protein-coding genes. The trees differed only in branch lengths (Figure 3). Monophyly of the Neobatrachia was supported by our work and previous studies [42, 43], while the Archaeobatrachia was paraphyletic [44, 45]. The major clades of frogs (Figure 3) were consistent in recent morphological and molecular analyses [46–48].

**Figure 4:**
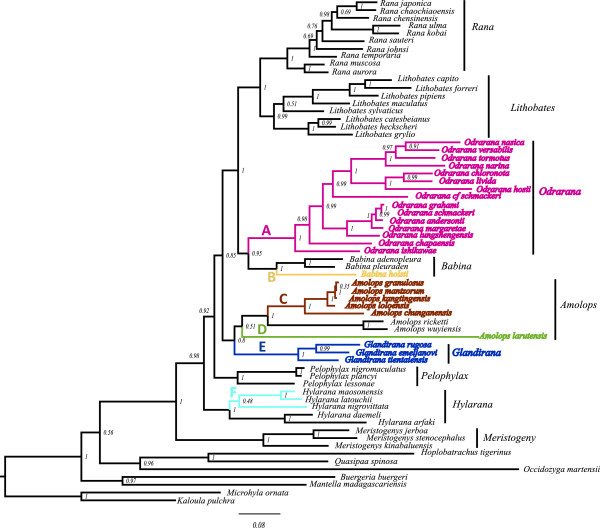
**A strict consensus tree from the Bayesian inference analyses derived from the sequences of the genes 12S and 16S rRNA for Ranidae.** Numbers above the lines or beside the nodes were Bayesian posterior probabilities. The mt genomic features of each species on the tree are as follows: **(A)** translocation of *trnH*; **(B)** *trnE* transposition and CR-psH-S1-*nad5*; **(C)** *WANCY* rearrangement; **(D)** transposition of *trnG*-*nad3* block and duplication of CR; **(E)** *trnS (AGY)* pseudogene; **(F)** translocation of *trnC*.

The aligned ranid data contained 1322 nucleotide positions. Of these sites, 677 were variable and 535 were potentially parsimony-informative. Figure 4 depicted the phylogeny of ranids based on 12S and 16S rRNA. ML and BI analyses produced identical trees. All neobatrachian families (Ranidae, Dicroglossidae, Rhacophoridae, Mantellidae, and Microhylidae) formed a clade and monophyly of each ranid genus was well supported. Within the Ranidae, our analyses recovered a sister taxa relationship between *Rana* + *Lithobates* and *Odorrana* + *Babina* (Figure 4), which was consistent previous studies [48]. However, the phylogenetic relationships among *Amolops*, *Glandirana*, *Pelophylax*, and *Hylarana* conflicted with other hypotheses [42, 49, 50]. Our results located *Glandirana* as the sister taxon of *Amolops*, but with weak Bayesian support (BPP = 80). Analyses of the mt protein-coding genes and the 12S and 16S rRNA (Figures 3, 4) did not support monophyly of section *Pelophylax* (including the subgenera *Pelophylax* and *Rugosa* [*Glandirana*]) as proposed by Dubois [51]. This corresponded to the previous view that *Pelophylax* was polyphyletic [50].

We mapped genomic features of neobatrachian mtDNA on the phylogeny (Figures 3, 4) to provide additional data for inferring history. Generally, gene rearrangements have been considered to be relatively rare, random events and, thus, they constituted useful synapomorphies [52–54]. Four tRNA genes, *trnL* (*CUN*), *trnT*, *trnP*, and *trnF*, were rearranged to form the *LTPF* cluster (labeled “A” in Figure 3), which was a synapomorphy for the Neobatrachia. Descendants shared unique gene rearrangements in ancestral lineages and/or additional rearrangements. For example, all the descendants of Dicroglossidae shared the *trnM* duplication [28, 33, 47, 55] (Figure 3). The Rhacophoridae and Mantellinae shared the translocation of *nad5* [56, 57] (Figure 3). Finally, the five species of *Amolops* formed a clade and all species shared the same *WANCY* gene rearrangement (Figure 4).

Gene rearrangements comprised distinct genomic characters for some genera (Figures 3, 4). For example, *Glandirana* was associated with genomic features derived from the pseudogene *trnS* (*AGY*), *Odorrana* was associated with structural *trnH* translocations, and *Pelophylax* retained the ancestral condition of typical *LTPF*. The derived features of the mitogenomes could serve as useful indicators of phylogenetic relationships, especially in lineages gene sequence data often lead to ambiguous results [42, 48]. Duplication of the *trnH*–*trnE* block supports the sister relationship of *Babina* and *Odorrana* [41] (Figure 4). Duplication of *trnM* also supports the rooting of *Occidozyga martensii* as the sister group of Dicroglossidae (Figure 3).

In highly rearranged lineages, convergent and parallel gene rearrangements happened frequently in non-sister lineages. Thus, genomic features require careful consideration when being employed for phylogenetic inference. Gene rearrangements vary in their phylogenetic distributions and rates among lineages [4]. In neobatrachians, convergent gene rearrangements occur. For example, a single origin cannot be invoked to explain the distribution of *trnM* in both Mantellinae and Dicroglossidae; the duplications arose independently (Figure 3) and the positions and residues of duplicated fragments differ [3, 28, 35]. A similar pattern involving the translocation of *nad5* was observed in the Rhacophoridae and *Fejervarya* [33, 56]. Parallel rearrangements also occur in gene rearrangement hotspots (e.g., *WANCY* and CR) [3].

### Correlation between codon usage and tRNA gene position

**Figure 5:**
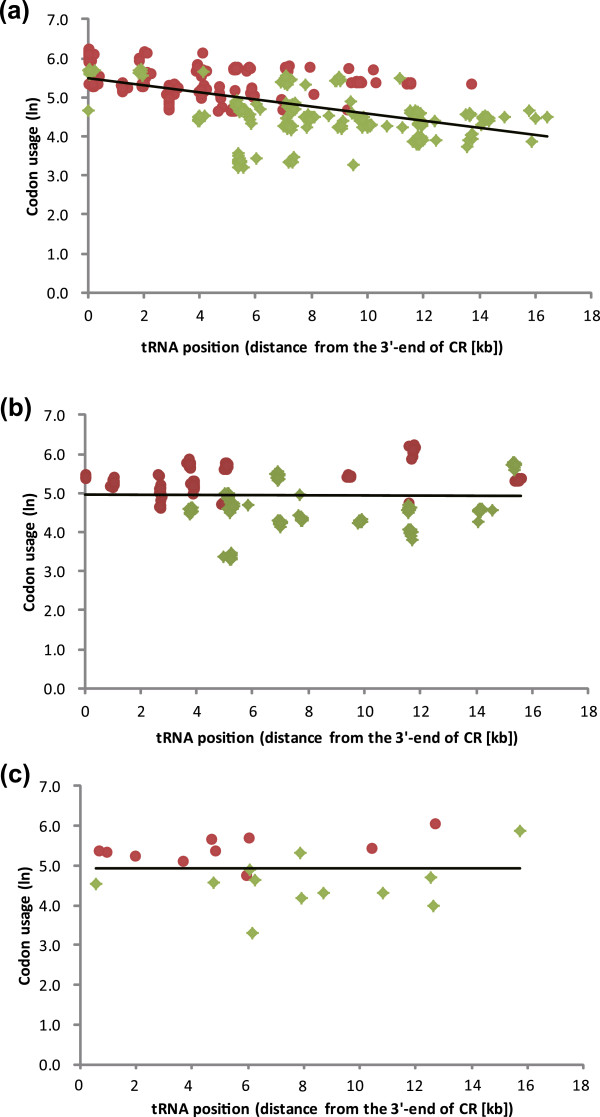
**A linear regression plot between position of tRNA genes and usage of the corresponding codon. (a)** 15 species with evolutionarily stable gene order; **(b)** 28 slightly rearranged gene orders in neobatrachians; **(c)** *Leiopelma archeyi*. Data points for the tRNA genes that specify hydrophobic and hydrophilic amino acids are colored magenta and green, respectively. The regression lines were derived from the all data points in each plot.

There are no significant correlations between codon usage and the location of tRNA in most of the AGOs, and no increased correlations in recurrent rearranged neobatrachians compared to basal neobatrachians (Additional file 3). Among the analyzed species, 14 have the AGOs of other vertebrates and 30 RGOs occur in *Leiopelma archeyi* and the neobatrachians (Additional file 2). Codon usage and tRNA position in the AGOs are very weakly correlated (Figure 5a; Pearson’s correlation coefficient *r* = -0.005, two-tailed *p* = 0.93, *n* = 308; Spearman’s rho correlation coefficient *r* = -0.118, two-tailed *p* = 0.039, *n* = 308). In contrast, the two variables show a significant correlation in neobatrachian RGOs (Figure 5b; Pearson’s correlation *r* = -0.551, two-tailed *p* < 0.001, *n* = 625; Spearman’s rho coefficient nonparametric correlation coefficient *r* = -0.607, two-tailed *p* < 0.001, *n* = 625). However, codon usage and tRNA positions are not significantly correlated in *L. archeyi*, which is an archaeobatrachian with an independent RGO (Figure 5c; Pearson’s correlation coefficient *r* = 0.001, two-tailed *p* = 0.998, *n* = 22; Spearman’s rho correlation coefficient *r* = -0.06, two-tailed *p* = 0.789, *n* = 22). Correlations between codon usage and tRNA do not increase in neobatrachians upon adding rearrangements relative to basal neobatrachians (Additional file 3). As seen in *Glandirana* and *Limnonectes bannaensis*, the loss of tRNA genes does not effect this correlation.

Codon usages and tRNA positions were thought to be significantly correlated in typical vertebrates [14]. The tRNA genes of these amino acids occur near the CR where transcription efficiency is high [13]. In mitogenomes, the 13 protein-coding genes associate with transmembrane proteins that are rich in hydrophobic amino acid residues. The tRNA genes that specify hydrophobic amino acid locate much closer to the CR than do hydrophilic amino acids. Consequently, some form(s) of adaptive selection might maintain novel mt gene orders and the rearrangement of genes with higher codon usage to regions near the CR to enhance transcription efficiency [14]. However, our analyses fail to detect a significant correlation in many archaeobatrachians possessing AGOs as well as in *Leiopelma archeyi* (Additional file 3). In comparison, significant correlations occur between codon usage and position of tRNAs in all neobatrachians sharing rearrangement *LTPF*. However, this does not infer a recent adaptation in the lineages with RGOs. Although rearrangement *LTPF* dramatically improves correlations, all other recurrent rearrangements do not do so. These recurrent rearrangements marginally or insignificantly improve correlations in neobatrachians (Additional file 3). Thus, highly-used tRNA genes in recent, re-appearing RGOs do not appear to be located closer to the CR than typical RGOs.

### Codon usage bias and tRNA gene number

Analyses of the relative synonymous codon usage (RSCU) in 14 anurans mitochondrial genomes yield insights into the relationship between changes in the number of tRNA genes (e.g., duplication/loss) and codon usage bias. The results did not indicate a difference in codon usage bias in duplicated or lost tRNA genes (Additional file 4). Gene *trnM* was duplicated in Dicroglossidae and *Mantella madagascariensis* yet their codon usage does not differ from those of other anurans (Chi-square test, *p* >0.05). Three species of *Glandirana* that lost *trnS (AGY)* did not differ in codon usage from closely related species (*p* >0.05). The same situation occurs in *Limnonectes bannaensis* in which tRNAs *trnA*, *trnN*, *trnC*, and *trnE* are absent [34].

The change in tRNA copy numbers could have accompanied gene rearrangement, as explained by the tandem duplication-random loss (TDRS) model [39, 58]. Directional mutation pressure on each strand of DNA and translational selection are major factors in codon usage bias [59]. Codon usage and tRNA abundance are tightly correlated in *Escherichia coli* and *Saccharomyces cerevisiae* [19]. This implies that tRNA gene copy numbers can evolve to match codon usage. Under translational selection, codon usage and tRNA gene content may have evolved to alternative stable states, where selection favors codons that are most rapidly translated by the current tRNAs [17]. However, tRNA gene duplications and losses do not show significant changes in codon bias among closely related anurans. The long period of accumulating mutations and the compactness of mtDNA might preclude such changes. The importation of nuclear tRNAs may also explain the absence of a correlation. Protein synthesis requires the replacement of tRNAs. In most organisms, mitochondrial biogenesis requires the importation of both large number of proteins and at least a few cytosolic tRNAs [20, 60]. Cytosolic tRNAs replace lost mt tRNA as well as reduce the influence of mt tRNA gene duplications or rearrangements. This alone may explain the absence of correlation between tRNA position and codon usage. Even though the importation of cytosolic tRNAs may compensate for missing mt tRNA genes, most vertebrates have a complete set of mt tRNA genes [20, 61]. The tRNA genes absent in *Glandirana* also occur rarely in other vertebrates, though they have been found in the wallaroo (*Macropus robustus*) [62], Chinese big-headed turtle *Platysternon megacephalum* [63] and large-headed frog *Limnonectes bannaensis* [34].

### Nucleotide substitution and mitogenomic reorganization

**Figure 6:**
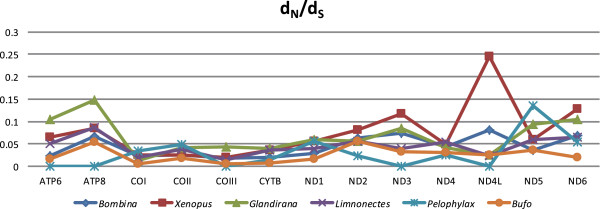
**Six pairwise comparison of sequence divergence for each mitochondrial gene.** Species-pairs are as follows: *Bombina maxima* and *B. bombina*; *Xenopus laevis* and *X. tropicalis*; *Glandirana emeljanovi* and *G. rugosa*; *Limnonectes fujianensis* and *L. bannaensis*; *Pelophylax nigromaculata* and *P. chosenica*; and *Bufo japonicas* and *B. gargarizans*.

Neobatrachian mt genomes have higher nucleotide substitution rates than archaeobatrachians [23, 37, 64]. To check the differences in the rates of nucleotide divergence in neobatrachians, we calculated average synonymous (d_S_) and nonsynonymous (d_N_) substitutions using a maximum likelihood tree for each of the 13 mitochondrial protein-coding genes as well as six congeneric pairwise comparisons among our anurans. Congeneric comparisons comprised two AGOs (*Bombina maxima* and *B. bombina*; and *Xenopus laevis*, and *X. tropicalis*) and four RGOs (*Glandirana emeljanovi* and *G. rugosa*; *Limnonectes fujianensis* and *L. bannaensis*; *Pelophylax nigromaculata* and *P. chosenica*; and *Bufo japonicas*, and *B. gargarizans*) (Figure 6). All neobatrachian mitochondrial genes have a significantly elevated d_N_ (*p* < 0.01) and d_S_ (*p* < 0.01).

Nonsynonymous/synonymous substitution rates (d_N_/d_S_ = ω) for each gene can test whether or not mitogenomic reorganization associates with an overall relaxation of selective pressures on mt function. In all comparisons, d_N_/d_S_ differs significantly from a null ratio of 1 (Figure 6; range 0.000–0.245). The mean d_N_/d_S_ does not differ significantly between the AGOs and RGOs (*p* >0.05). ML estimates of ω indicate that purifying selection acts on each protein-coding gene; no significant difference (*p* >0.05) occurs in the fitting of models M1a (nearly neutral) and M2a (positive selection) (Additional file 5). Estimates of ω under the two-ratio model for the Anura are not higher than its null model and only *cob* differs significantly by LRT (Table 2, Additional file 5). Values of ω are significantly less than 1 for all 13 protein-coding genes in the AGO and RGOs (Figure 6, Table 2). This result supports the presence of strong purifying selection in anurans [23, 37], which strongly preserves mt gene function [65, 66]. However, ratios of ω for all genes, except *cob*, *nad3*, *nad4L*, and *nad6*, are significantly higher in neobatrachians when compared to non-neobatrachians (Table 2). Similarly, independent values of ω inferred for the stem branch of Natatanura are generally higher than the respective null model and evaluations of most individual genes obtain significant results (Table 2, Additional file 5). Corresponding to previous studies [37], the elevated ω in the branch leading to the Neobatrachia indicates the possible relaxation of purifying selection on protein-coding genes (Table 2).

**Table 2:** **Results from branch models that assume branch-specific changes in the selection coefficient (ω) in Neobatrachia, based on single-gene alignments and the all-mt gene datasets**

Models gene	Null model	Neobatrachia		Anura		Natatanura	
		Backgr	Branch	Backgr	Branch	Backgr	Branch
*atp6*	0.0349	**0.0186**	**0.0433**	0.0166	0.0354	**0.0193**	**0.0570**
*atp8*	0.0399	-	-	-	-	-	-
*cox1*	0.0108	**0.0072**	**0.0138**	0.0078	0.0111	**0.0077**	**0.0163**
*cox2*	0.0266	**0.0186**	**0.0330**	0.0205	0.0270	**0.0184**	**0.0401**
*cox3*	0.0270	**0.0226**	**0.0298**	0.0344	0.0265	**0.0228**	**0.0327**
*cob*	0.0277	0.0294	0.0268	**0.0519**	**0.0265**	0.0256	0.0303
*nad1*	0.0331	**0.0262**	**0.0376**	0.0364	0.0329	**0.0266**	**0.0427**
*nad2*	0.0560	**0.0457**	**0.0607**	0.0282	0.0571	**0.0437**	**0.0702**
*nad3*	0.0618	0.0615	0.0619	0.0550	0.0624	0.0534	0.0730
*nad4*	0.0432	**0.0367**	**0.0470**	0.0201	0.0439	**0.0362**	**0.0527**
*nad4L*	0.0369	0.0435	0.0335	0.0270	0.0371	0.0377	0.0362
*nad5*	0.0479	**0.0385**	**0.0552**	0.0428	0.0482	**0.0382**	**0.0663**
*nad6*	0.0370	0.0308	0.0393	0.0192	0.0381	0.0336	0.0411
All gene	0.0480	**0.0428**	**0.0516**	0.0494	0.0479	**0.0410**	**0.0584**

Bold highlight results that are significantly different to the null model (LRT *p* < 0.05).

A positive correlation occurs between the rates of mt gene rearrangement and mt nucleotide diversity in some lineages of invertebrates [21, 67, 68]. Accordingly, Shao et al. [67] hypothesized that an increase in substitution rates can drive elevated rates of gene rearrangement. A high substitution rate leads to an increase in mutation at the sites of initiation and termination of the mt genome replication. These mutations may cause errors during replication of mt genomes and then cause gene rearrangements through gene duplications and deletions [15, 38]. Considering the TDRL model, a high rate of nucleotide substitution leads to an increase in the rate of mt gene rearrangement. High rates of nucleotide substitution also tend to occur in modified genomes of salamanders [69]. However, fast nucleotide substitution rates are not required to increase the propensity of mitogenomic rearrangements [23, 37]. Our results show that neobatrachian mt genes have a significantly elevated d_N_ (*p* < 0.01) and d_S_ (*p* < 0.01). Irisarri et al. [23] proposed that an accelerated rate of mt substitution rate was a result of the relaxation of purifying selection on protein-coding genes. In neobatrachians, some clades, such as Rhacophoridae, Mantellinae, and Dicroglossidae (Figure 3), have an increase in the rate of mt gene rearrangement. However, no significant difference in substitution rate occurs between non-rearranged and rearranged ranoids [37]. Thus, the high diversity of recurrent rearrangements in neobatrachians appears to be driven by variables beyond a high rate of nucleotide substitution, for example, life histories and genetic drift—both of which may increase the chance that rearranged mt genomes survive and drive fixation [67]. Considering rearrangements in the archaeobatrachians *Leiopelma archeyi* and *Leptolalax pelodytoides* [24, 27], accelerated substitution rates may be lineage-specific features, rather than accompanying gene rearrangement. No differences in rate of change occur between lineages with gene duplications and their non-duplicated counterparts [37]. Our results also failed to detect significant differences in rates when tRNA genes are lost (e.g., *Glandirana* branch, LRT *p* >0.05). Overall, these results indicate that high substitution rates and the relaxation of purifying selection can increase the chance of mitogenomic reorganization in neobatrachians, but they are not required.

### Selective pressure on mitogenomic reorganization

Selection on reorganized mitogenomes is less well studied compared to selection on mt protein-coding genes [2, 14]. Although a positive correlation might occur between codon usage and tRNA gene positions [14], genomic reorganization does not improve this relationship. Adaptive selection does not appear to act on tRNA gene positions after genomic rearrangement. Further, the widespread importation of cytosolic tRNA into mitochondria [20] may preclude a codon usage bias owing to tRNA gene duplications or losses. The nonadaptive evolution of gene order suggests that random loss follows gene duplication. Hotspots of gene rearrangement (eg. *WANCY*) in *Amolops* and *Odorrana* (Figure 3) have similar portions of duplicated genes yet the positions of silenced genes vary. These findings support the TDRL model of rearrangement in many neobatrachians [22, 29, 56].

Nucleotide substitution rates and ω are higher in neobatrachian mt genomes than in archaeobatrachians and yet no significant correlation exists between the rates of mitogenomic reorganization and mt nucleotide diversity [23, 37]. Values of ω strongly indicate that purifying selection likely contributes to the maintenance of metabolic function in anurans and trans-membrane protein functions likely constrain nonsynonymous mutations [23]. Some functional constraints on the mitogenomic organization of rRNA and tRNA genes exist (e.g., 12S and 16S rRNAs generally locate near the CR due to requiring high transcriptional rates [13]; secondary structures of tRNA genes involve punctuation models or termination signals [70]). Functional constraints do not necessarily favor one gene rearrangement over another. The fixation of large-scale mitogenomic reorganization largely involves two nonadaptive fores: random genetic drift and mutation pressure [11, 71]. Further studies on the molecular and demographic mechanisms of this hypothesis may yield new insights into the evolution of mitogenomic reorganization.

## Methods

We sequenced the mt genome of *Glandirana rugosa*, *G. emeljanovi*, and *G. tientaiensis* (Table 1). We also sequenced four mtDNA fragments including one conserved sequence [12S–16S rRNA] and three hotspots of rearrangements in 20 ranid species across the genera *Amolops*, *Babina*, *Hylarana*, *Odorrana* and *Rana* (Table 1). Specimens were stored in 95% ethanol at -20°C in the Chengdu Institute of Biology. All work with animals was conducted according to relevant national and international guidelines. All animal care and experimental procedures were approved by the Chengdu Institute of Biology Animal Care and Use Committee.

Total genomic DNA was extracted from muscle tissue through SDS-proteinase K/phenol-chloroform protocols [72]. Using complete mtDNA information available for Ranidae, we designed 13 pairs of primers (Additional file 6) that amplified overlapping fragments spanning the entire mt genome. From our 20 species, we determined three hotspot fragments (*nad2*–*cox1*, *nad3*–*nad5*, and *nad5*–*cob*) and partial fragments of 12S and 16S rRNA genes using primers described in Kurabayashi [73]. We amplified these fragments using a combination of normal polymerase chain reaction (PCR) and long-and-accurate (LA) PCR methods; normal PCR was carried out in a 25 μL mixture containing 0.5–1.0 μL of template DNA, 2.5 μL 10 × PCR buffer, 0.5 μL of each primer (10 pm/μL), 2 μL MgCl_2_, 2 μL dNTPs and 0.3 μL of Extaq DNA polymerase (TaKaRa Bio, Dalian, China) with reaction conditions as follows: 4 min at 95°C, followed by 33 cycles of 40 s at 94°C, 45 s at 48–58°C, 1.5 min at 72°C and a final extension of 8 min at 72°C. LA-PCR was carried out in a 25 μL mixture containing 0.5–1.0 μL of template DNA, 12.5 μL 2 × PCR buffer, 0.5 μL of each primer (10 pm/μL), 4 μL dNTPs and 0.5 μL of KOD FX DNA polymerase (TOYOBO, Osaka, Japan). PCR conditions consisted of 3 min at 98°C, followed by 33 cycles of 50 s at 94°C, 60 s at 50–58°C, 4 min at 72°C and a final extension for 10 min at 72°C. We sub-cloned PCR fragments of the CR containing long tandem repeats using a pMD™ 18-T Vector (TaKaRa Bio, Dalian, China) and sequenced this fragment by additional walking primers (Additional file 6). DNA sequencing was performed on an ABI 3730 automatic DNA sequencer.

### Genome annotation

We extracted protein-coding sequences from each mt genome and identified mt tRNA genes using tRNAscan-SE v.1.21 (http://lowelab.ucsc.edu/tRNAscan-SE) [74]. We excluded incorrect annotations by comparing original annotations from GenBank with the vertebrate mt genetic code. We identified *trnS (AGY)* by visually inspecting unassigned regions for sequences with similarity and assigning them to previously identified mt tRNA isotypes. We determined the locations of the 13 protein-coding and 2 rRNA genes through comparisons with homologous sequences in other anurans [22, 29, 30].

We downloaded 40 complete and 3 partial anuran mt genomes from GenBank (Additional file 2). We used taxonomic names prior to Frost [75] to test their hypothesis and referred to NCBI Organelle Genome Resource and MitoZoa (http://www.caspur.it/mitozoa) [76] to determine genome organizations for each species (Additional file 2).

### Measuring codon usage and the position of each tRNA gene

We chose 15 archaeobatrachian species plus 29 neobatrachian species to examine variation in codon usage and gene arrangement. We determined correlations between physical (base pair [bp]) distances of each tRNA gene from the CR (from the 5’ end of the tRNA gene to the 3’ end of the CR) and codon usage of the 13 protein-coding genes (where overlapping codons were considered once for each gene) following Satoh et al. [14]. For mt genomes with two CR or tRNA copies, we analyzed the first copy.

To evaluate the codon usage bias in a single codon family, we calculated relative synonymous codon usage (RSCU) values of every codon in each sample. RSCU values were obtained by using DAMBE [77] and MEGA5 [78]. We evaluated *trnL* and *trnS* codons, which had two groups of codons (CUN and UUR, and AGY and UCN, respectively), as two synonymous codon families [79].

### Genetic divergence, molecular evolution and phylogenetics

We aligned sequences of the 13 mt protein-coding genes using ClustalW in MEGA5 [78]. To avoid artificial alignment errors, we used Gblocks v.0.19b [80] with the following settings to remove ambiguous alignments: 26 minimum conserved positions, 42 minimum flanking positions, 8 maximum non-conserved positions, and a minimum length of a block of 5 while allowing gap positions in half.

We used DnaSP 5.10 [81] to determine DNA polymorphism and divergence, including the number of synonymous substitutions per synonymous site (d_S_) and nonsynonymous substitutions per nonsynonymous site (d_N_). Maximum likelihood (ML) estimates of nonsynonymous/synonymous substitution rates (ω) were obtained with CODEML in the PAML4.4 package [82]. The nearly neutral (M1a) and positive selection (M2a) models were compared using a likelihood ratio test (LRT). To detect if the 13 protein-coding genes of neobatrachian species experienced divergent patterns of selection compared to non-neobatrachian species and other amphibians, we applied a two-ratio model test to the each-gene and all-gene datasets. Two separate two-ratio models were fitted: one ratio was assigned to the interested branch (Anura, Neobatrachia, Natatanura) and the other was assigned to the remaining lineages. These two-ratio models were also compared against the null model (one ratio model) by LRT.

To further confirm the phylogenetic relationships among anurans, 46 Anura and four outgroup taxa (two Caudata [GenBank: NC_008085, NC_004926] and two Gymnophiona [GenBank: NC_006301, NC_006302]) were included in this analyses. We constructed the phylogenies using the concatenated 13 mt protein-coding genes and partitioned these genes by codon position. The best fitted substitution model for each partition was estimated using Akaike information criterion (AIC) implemented in jModeltest [83]. The model of GTR + I + G was chosen for ML and Bayesian inference (BI) analyses, which were performed with RAxML BlackBox web-servers (http://phylobench.vital-it.ch/raxml-bb/index.php) [84] and MrBayes 3.1 [85], respectively. BI analyses used the following settings: 10 million Markov chain Monte Carlo (MCMC) generations, a sampling frequency of 1000, and calculating a majority rule consensus tree after omitting the first 25% trees as burn-in.

To provide further insight into the phylogenetic relationships of species with gene rearrangments, we conducted additional ML and BI analyses using 12S and 16S rRNA sequence data from 65 taxa (58 ranids from nine genera, three dicroglossids, one rhacophorid, one mantellid, and two microhylids; Figure 4). Alignment of these sequences was verified using secondary structure [86]. We used the same procedures for tree reconstruction as described above for the 13 mt protein-coding genes.

### Availability of supporting data

Organization and features of mitochondrial genome in three species of *Glandirana* are available in Additional file 1. Detailed information of 46 anurans mitochondrial genomes used in this study is given in Additional file 2. The correlations between the codon usage and tRNA positions of each 44 anuran species are available in Additional file 3. Relative synonymous codon usage (RSCU) values in 13 protein-coding genes of anurans mitochondrial genomes are given in Additional file 4. Positive selection detection of the mt protein genes among different branches are presented in Additional file 5. Primers designed for amplifying the complete mitochondrial genome of *Glandirana* are listed in Additional file 6.

## Electronic supplementary material

Additional file 1: **Organization and features of mitochondrial genome in three species of** ***Glandirana.*** (DOCX 30 KB)

Additional file 2: **Gene organization of 46 anurans mitochondrial genomes.** GenBank accession numbers and classification of anuran mitochondrial genomes used in this study. Organizations for each species were checked with NCBI Organelle Genome Resource and MitoZoa (http://www.caspur.it/mitozoa). Gene nomenclature from MitoZoa. (XLS 66 KB)

Additional file 3: **The correlation between the codon usage and tRNA positions of each 44 anuran species.** (DOCX 248 KB)

Additional file 4: **Relative synonymous codon usage (RSCU) values in 13 protein-coding genes of anurans mitochondrial genomes.** (XLS 46 KB)

Additional file 5: **Positive selection detection of the mt protein genes among different branches.** (DOCX 18 KB)

Additional file 6: **Primers designed for amplifying the complete mitochondrial genome of** ***Glandirana***. (DOCX 17 KB)

## Abbreviations

atp6: ATP synthase subunit 6
atp8: ATP synthase subunit 8
cob: Cytochrome *b*
cox1: Cytochrome c oxidase subunit I
cox2: Cytochrome c oxidase subunit II
cox3: Cytochrome c oxidase subunit III
CSB: Conserved sequence blocks
CR: Control regions
ESGO: Evolutionarily stable gene orders
LTPF: *trnL (CUN)*, *trnT*, *trnP*, and *trnF*
LRT: Likelihood ratio test
mt: Mitochondrial
nad1: NADH dehydrogenase subunit 1
nad2: NADH dehydrogenase subunit 2
nad3: NADH dehydrogenase subunit 3
nad4: NADH dehydrogenase subunit 4
nad4L: NADH dehydrogenase subunit 4 L
nad5: NADH dehydrogenase subunit 5
nad6: NADH dehydrogenase subunit 6
OL: The origin of light strand replication
O_H_: The origin of heavy strand replication
RGO: Rearranged gene orders
TAS: Termination associated sequence
TDRL: Tandem duplication and random loss
WANCY: *trnW*, *trnA*, *trnN*, OL, *trnC*, and *trnY*.
