# Supplementary material for: The evolution of mitochondrial genomes in modern frogs (Neobatrachia): nonadaptive evolution of mitochondrial genome reorganization
Source: BMC Genomics. 2014 Aug 20;15(1):691. doi: 10.1186/1471-2164-15-691 (PMC4153901; doi:10.1186/1471-2164-15-691)
Supplement: Supplementary file 1 — Additional file 1: Organization and features of mitochondrial genome in three species of Glandirana. (DOCX 30 KB) [file 12864_2013_6391_MOESM1_ESM.docx]

Table S1 Organization and features of *Glandirana rugosa* mitochondrial genome

| **Gene/region** | **Position** | **Spacer(+)**  **Overlap(-)** | **Size**  **(bp)** | **strand** | **codon** | |
| --- | --- | --- | --- | --- | --- | --- |
|  |  |  |  |  | **start** | **stop** |
| ***trnL (CUN)*** | **1-72** |  | **72** | **H** |  |  |
| ***trnT*** | **75-145** | **+2** | **71** | **H** |  |  |
| ***trnP*** | **138-206** | **-8** | **69** | **L** |  |  |
| ***trnF*** | **208-276** | **+1** | **69** | **H** |  |  |
| ***12S rRNA*** | **277-1208** |  | **932** | **H** |  |  |
| ***trnV*** | **1209-1278** |  | **70** | **H** |  |  |
| ***16S rRNA*** | **1279-2865** |  | **1587** | **H** |  |  |
| ***trnL(UUR)*** | **2866-2938** |  | **73** | **H** |  |  |
| ***nad1*** | **2940-3900** | **+1** | **961** | **H** | **ATG** | **T** |
| ***trnI*** | **3901-3971** |  | **71** | **H** |  |  |
| ***trnQ*** | **3972-4042** |  | **71** | **L** |  |  |
| ***trnM*** | **4042-4110** | **-1** | **69** | **H** |  |  |
| ***nad2*** | **4111-5145** |  | **1035** | **H** | **ATT** | **TAG** |
| ***trnW*** | **5144-5213** | **-2** | **70** | **H** |  |  |
| ***trnA*** | **5214-5282** |  | **69** | **L** |  |  |
| ***trnN*** | **5283-5355** |  | **73** | **L** |  |  |
| **O_L_** | **5356-5383** |  | **28** | **H** |  |  |
| ***trnC*** | **5381-5444** | **-3** | **64** | **L** |  |  |
| ***trnY*** | **5445-5511** |  | **67** | **L** |  |  |
| ***cox1*** | **5516-7066** | **+4** | **1551** | **H** | **ATA** | **AGG** |
| ***trnS(UCN)*** | **7058-7128** | **-9** | **71** | **L** |  |  |
| ***trnD*** | **7130-7198** | **+1** | **69** | **H** |  |  |
| ***cox2*** | **7199-7886** |  | **688** | **H** | **ATG** | **T** |
| ***trnK*** | **7887-7955** | **-2** | **69** | **H** |  |  |
| ***atp8*** | **7957-8121** |  | **165** | **H** | **ATG** | **TAA** |
| ***atp6*** | **8112-8793** | **-10** | **682** | **H** | **ATG** | **T** |
| ***cox3*** | **8794-9577** |  | **784** | **H** | **ATG** | **T** |
| ***trnG*** | **9578-9646** |  | **69** | **H** |  |  |
| ***nad3*** | **9647-9986** |  | **340** | **H** | **ATG** | **T** |
| ***trnR*** | **9987-10055** |  | **69** | **H** |  |  |
| ***nad4L*** | **10056-10340** |  | **285** | **H** | **ATG** | **TAA** |
| ***nad4*** | **10334-11693** | **-6** | **1360** | **H** | **ATG** | **T** |
| ***trnH*** | **11694-11762** |  | **69** | **H** |  |  |
| ***Ψ (trnS)*** | **11763-11824** |  | **61** | **H** |  |  |
| ***nad5*** | **11857-13645** |  | **1789** | **H** | **ATG** | **T** |
| ***nad6*** | **13643-14140** | **-3** | **498** | **L** | **ATG** | **AGA** |
| ***trnE*** | **14142-14210** | **+1** | ***69*** | **L** |  |  |
| ***cob*** | **14213-15355** | **+2** | **1143** | **H** | **ATG** | **TAG** |
| **D-loop** | **15356-** |  |  | **H** |  |  |

Table S2 Organization and features of *Glandirana emeljanovi* mitochondrial genome

| **Gene/region** | **Position** | **Spacer(+)**  **Overlap(-)** | **Size**  **(bp)** | **strand** | **codon** | |
| --- | --- | --- | --- | --- | --- | --- |
|  |  |  |  |  | **start** | **stop** |
| ***trnL (CUN)*** | **1-72** |  | **72** | **H** |  |  |
| ***trnT*** | **75-145** | **+3** | **71** | **H** |  |  |
| ***trnP*** | **138-206** |  | **69** | **L** |  |  |
| ***trnF*** | **208-276** |  | **69** | **H** |  |  |
| ***12S rRNA*** | **277-1209** |  | **933** | **H** |  |  |
| ***trnV*** | **1210-1279** |  | **70** | **H** |  |  |
| ***16S rRNA*** | **1280-2863** |  | **1584** | **H** |  |  |
| ***trnL(UUR)*** | **2864-2936** |  | **73** | **H** |  |  |
| ***nad1*** | **2938-3898** | **+1** | **961** | **H** | **ATG** | **T** |
| ***trnI*** | **3899-3969** |  | **71** | **H** |  |  |
| ***trnQ*** | **3970-4040** |  | **71** | **L** |  |  |
| ***trnM*** | **4040-4108** |  | **69** | **H** |  |  |
| ***nad2*** | **4109-5143** |  | **1035** | **H** | **ATT** | **TAG** |
| ***trnW*** | **5142-5211** | **-2** | **70** | **H** |  |  |
| ***trnA*** | **5212-5280** |  | **69** | **L** |  |  |
| ***trnN*** | **5281-5353** |  | **73** | **L** |  |  |
| **O_L_** | **5354-5381** | **-3** | **28** | **H** |  |  |
| ***trnC*** | **5379-5442** |  | **64** | **L** |  |  |
| ***trnY*** | **5443-5509** |  | **67** | **L** |  |  |
| ***cox1*** | **5514-7064** | **+4** | **1551** | **H** | **ATA** | **AGG** |
| ***trnS(UCN)*** | **7056-7126** | **-9** | **71** | **L** |  |  |
| ***trnD*** | **7128-7196** | **+1** | **69** | **H** |  |  |
| ***cox2*** | **7197-7884** |  | **688** | **H** | **ATG** | **T** |
| ***trnK*** | **7885-7953** | **-2** | **69** | **H** |  |  |
| ***atp8*** | **7955-8119** |  | **165** | **H** | **ATG** | **TAG** |
| ***atp6*** | **8110-8791** | **-10** | **682** | **H** | **ATG** | **T** |
| ***cox3*** | **8792-9575** |  | **784** | **H** | **ATG** | **T** |
| ***trnG*** | **9576-9644** |  | **69** | **H** |  |  |
| ***nad3*** | **9645-9984** |  | **340** | **H** | **ATG** | **T** |
| ***trnR*** | **9985-10054** |  | **70** | **H** |  |  |
| ***nad4L*** | **10055-10339** |  | **285** | **H** | **ATG** | **TAA** |
| ***nad4*** | **10333-11692** | **-6** | **1360** | **H** | **ATG** | **T** |
| ***trnH*** | **11693-11761** |  | **69** | **H** |  |  |
| ***Ψ (trnS)*** | **11762-11824** |  | **62** | **H** |  |  |
| ***nad5*** | **11857-13645** |  | **1789** | **H** | **ATG** | **T** |
| ***nad6*** | **13643-14140** | **-3** | **498** | **L** | **ATG** | **AGA** |
| ***trnE*** | **14141-14208** |  | **68** | **L** |  |  |
| ***cob*** | **14211-15353** | **+2** | **1143** | **H** | **ATG** | **TAG** |
| **D-loop** | **15354-** |  |  | **H** |  |  |

Table S3 Organization and features of *Glandirana tientaiensis* mitochondrial genome

| **Gene/region** | **Position** | **Spacer(+)**  **Overlap(-)** | **Size**  **(bp)** | **strand** | **codon** | |
| --- | --- | --- | --- | --- | --- | --- |
|  |  |  |  |  | **start** | **stop** |
| ***trnL (CUN)*** | **1-72** |  | **72** | **H** |  |  |
| ***trnT*** | **75-144** | **+3** | ***70*** | **H** |  |  |
| ***trnP*** | **137-205** |  | **69** | **L** |  |  |
| ***trnF*** | **205-273** |  | **69** | **H** |  |  |
| ***12S rRNA*** | **274-1205** |  | **932** | **H** |  |  |
| ***trnV*** | **1206-1275** |  | **70** | **H** |  |  |
| ***16S rRNA*** | **1276-2858** |  | **1583** | **H** |  |  |
| ***trnL(UUR)*** | **2859-2931** |  | **73** | **H** |  |  |
| ***nad1*** | **2933-3893** |  | **961** | **H** | **ATG** | **T** |
| ***trnI*** | **3894-3964** |  | **71** | **H** |  |  |
| ***trnQ*** | **3964-4034** |  | **71** | **L** |  |  |
| ***trnM*** | **4034-4102** |  | **69** | **H** |  |  |
| ***nad2*** | **4103-5137** |  | **1035** | **H** | **ATT** | **TAG** |
| ***trnW*** | **5136-5205** |  | **70** | **H** |  |  |
| ***trnA*** | **5206-5275** |  | ***70*** | **L** |  |  |
| ***trnN*** | **5276-5348** |  | **73** | **L** |  |  |
| **O_L_** | **5349-5376** | **-3** | **28** | **H** |  |  |
| ***trnC*** | **5374-5437** |  | **64** | **L** |  |  |
| ***trnY*** | **5438-5504** |  | **67** | **L** |  |  |
| ***cox1*** | **5509-7059** | **+4** | **1551** | **H** | **ATA** | **AGG** |
| ***trnS(UCN)*** | **7051-7121** | **-9** | **71** | **L** |  |  |
| ***trnD*** | **7123-7191** | **+1** | **69** | **H** |  |  |
| ***cox2*** | **7192-7879** |  | **688** | **H** | **ATG** | **T** |
| ***trnK*** | **7880-7948** | **-2** | **69** | **H** |  |  |
| ***atp8*** | **7950-8114** |  | **165** | **H** | **ATG** | **TAA** |
| ***atp6*** | **8105-8786** | **-10** | **682** | **H** | **ATG** | **T** |
| ***cox3*** | **8787-9570** |  | **784** | **H** | **ATG** | **T** |
| ***trnG*** | **9571-9639** |  | **69** | **H** |  |  |
| ***nad3*** | **9640-9979** |  | **340** | **H** | **GTG** | **T** |
| ***trnR*** | **9980-10048** |  | **69** | **H** |  |  |
| ***nad4L*** | **10049-10333** |  | **285** | **H** | **ATG** | **TAA** |
| ***nad4*** | **10327-11686** | **-6** | **1360** | **H** | **ATG** | **T** |
| ***trnH*** | **11687-11754** |  | ***68*** | **H** |  |  |
| ***Ψ (trnS)*** | **11755-11817** |  | **62** | **H** |  |  |
| ***nad5*** | **11850-13638** |  | **1789** | **H** | **ATG** | **T** |
| ***nad6*** | **13636-14130** | **-3** | **495** | **L** | **ATG** | **AGA** |
| ***trnE*** | **14132-14200** | **+1** | ***69*** | **L** |  |  |
| ***cob*** | **14203-15345** | **+2** | **1143** | **H** | **ATG** | **TAG** |
| **D-loop** | **15346-** |  |  | **H** |  |  |

Table S4 Compositional features of protein-coding mitochondrial genes of *Glandirana*

| Feature | *G*. *rugosa* | | *G*. *emeljanovi* | | *G*. *tientaiensis* | |
| --- | --- | --- | --- | --- | --- | --- |
| Total length | 11274 |  | 11271 |  | 11268 |  |
| %A | 25.6 |  | 25.5 |  | 24.9 |  |
| %G | 14.2 |  | 14.5 |  | 14.6 |  |
| %C | 28.5 |  | 28.9 |  | 30.1 |  |
| %T | 31.6 |  | 31.1 |  | 30.3 |  |
| *atp6* | 682 | (ATG/T) | 682 | (ATG/T) | 682 | (ATG/T) |
| *atp8* | 165 | (ATG/TAG) | 165 | (ATG/TAA) | 165 | (ATG/TAA) |
| *cox1* | 1551 | (ATA/AGG) | 1551 | (ATA/AGG) | 1551 | (ATA/AGG) |
| *cox2* | 688 | (ATG/T) | 688 | (ATG/T) | 688 | (ATG/T) |
| *cox3* | 784 | (ATG/T) | 784 | (ATG/T) | 784 | (ATG/T) |
| *cob* | 1143 | (ATG/TAG) | 1143 | (ATG/TAG) | 1143 | (ATG/TAG) |
| *nad1* | 961 | (ATG/T) | 961 | (ATG/T) | 961 | (ATG/T) |
| *nad2* | 1035 | (ATT/TAG) | 1035 | (ATT/TAG) | 1035 | (ATT/TAG) |
| *nad3* | 340 | (ATG/T) | 340 | (ATG/T) | 340 | (GTG/T) |
| *nad4* | 1360 | (ATG/T) | 1360 | (ATG/T) | 1360 | (ATG/T) |
| *nad4L* | 285 | (ATG/TAA) | 285 | (ATG/TAA) | 285 | (ATG/TAA) |
| *nad5* | 1789 | (ATG/T) | 1789 | (ATG/T) | 1789 | (ATG/T) |
| *nad6* | 498 | (ATG/AGA) | 495 | (ATG/AGA) | 492 | (ATG/AGA) |
