# Supplementary material for: The evolution of mitochondrial genomes in modern frogs (Neobatrachia): nonadaptive evolution of mitochondrial genome reorganization
Source: BMC Genomics. 2014 Aug 20;15(1):691. doi: 10.1186/1471-2164-15-691 (PMC4153901; doi:10.1186/1471-2164-15-691)
Supplement: Supplementary file 3 — Additional file 3: The correlation between the codon usage and tRNA positions of each 44 anuran species. (DOCX 248 KB) [file 12864_2013_6391_MOESM3_ESM.docx]

Additional file 3

The correlation between the codon usage and tRNA positions of each 44 anuran species.

**Archaeobatrachia**

*Ascaphus truei Alytes obstetricans*

*n*= 22, *r*=-0.036, *p*= 0.873

*n*= 22, *r*= -0.056, *p*= 0.804

*Bombina bombina Bombina maxima*

*n*= 22, *r*=-0.015, *p*= 0.948

*n*= 22, *r*=-0.014, *p*= 0.951

*Bombina orientalis Bombina variegata*

*n*= 22, *r*=0.016, *p*= 0.942

*n*= 22, *r*=0.013, *p*= 0.955

*Discoglossus galganoi Hymenochirus boettgeri*

*n*= 22, *r*=0.012, *p*= 0.958

*n*= 22, *r*=-0.006, *p*= 0.979

*Leiopelma archeyi Pelobates cultripes*

*n*= 22, *r*=-0.037, *p*= 0.872

*n*= 22, *r*=0.001, *p*= 0.998

*Pipa carvalhoi Pseudhymenochirus merlini*

*n*= 22, *r*=-0.020, *p*= 0.930

*n*= 22, *r*=0.023, *p*= 0.919

*Rhinophrynus dorsalis Xenopus laevis*

*n*= 22, *r*=-0.058, *p*= 0.797

*n*= 22, *r*=0.017, *p*= 0.939

*Xenopus tropicalis*

*n*= 22, *r*=0.015, *p*= 0.948

**Neobatrachia**

*Bufo gargarizans Bufo japonicas*

*n*= 22, *r*=-0.568, *p*= 0.006

*n*= 22, *r*=-0.566, *p*= 0.006

*Bufo melanostictus Buergeria buergeri*

*n*= 22, *r*=-0.574, *p*= 0.005

*n*= 22, *r*=-0.595, *p*= 0.003

*Euphlyctis hexadactylus Fejervarya cancrivora*

*n*= 22, *r*=-0.555, *p*= 0.007

*n*= 22, *r*=-0.551, *p*= 0.008

*Fejervarya limnocharis Glandirana rugosa*

*n*= 21, *r*=-0.495, *p*= 0.023

*n*= 22, *r*=-0.567, *p*= 0.006

*Glandirana emeljanovi Glandirana tientaiensis*

*n*= 21, *r*=-0.506, *p*= 0.019

*n*= 21, *r*=-0.493, *p*= 0.023

*Hyla chinensis Hyla japonica*

*n*= 22, *r*=-0.564, *p*= 0.006

*n*= 22, *r*=-0.566, *p*= 0.006

*Hoplobatrachus tigerinus Kaloula pulchra*

*n*= 22, *r*=-0.568, *p*= 0.006

*n*= 22, *r*=-0.597, *p*= 0.003

*Limnonectes bannaensis Limnonectes fragilis*

*n*= 16, *r*=-0.761, *p*= 0.001

*n*= 18, *r*=-0.542, *p*= 0.020

*Limnonectes fujianensis Microhyla heymonsi*

*n*= 22, *r*=-0.559, *p*= 0.007

*n*= 22, *r*=-0.543, *p*= 0.009

*Microhyla okinavensis Microhyla ornate*

*n*= 22, *r*=-0.557, *p*= 0.007

*n*= 22, *r*=-0.558, *p*= 0.007

*Nanorana pleskei Occidozyga martensii*

*n*= 22, *r*=-0.599, *p*= 0.003

*n*= 22, *r*=-0.571, *p*= 0.006

*Odorrana tormotus Odorrana ishikawae*

*n*= 22, *r*=-0.544, *p*= 0.009

*n*= 22, *r*=-0.550, *p*= 0.008

*Pelophylax nigromaculata Pelophylax plancyi*

*n*= 22, *r*=-0.557, *p*= 0.007

*n*= 22, *r*=-0.563, *p*= 0.006

*Pelophylax chosenica Quasipaa spinosa*

*n*= 22, *r*=-0.582, *p*= 0.004

*n*= 22, *r*=-0.558, *p*= 0.007

*Rhacophorus schlegelii*

*n*= 22, *r*=-0.603, *p*= 0.003
