# Supplementary material for: The evolution of mitochondrial genomes in modern frogs (Neobatrachia): nonadaptive evolution of mitochondrial genome reorganization
Source: BMC Genomics. 2014 Aug 20;15(1):691. doi: 10.1186/1471-2164-15-691 (PMC4153901; doi:10.1186/1471-2164-15-691)
Supplement: Supplementary file 5 — Additional file 5: Positive selection detection of the mt protein genes among different branches. (DOCX 18 KB) [file 12864_2013_6391_MOESM5_ESM.docx]

Additional file 5

Positive selection detection of the mt protein genes among different branches.

| Models  gene | model =0  one-ratio | NSsites=1  NearlyNeutral | NSsites=2  Positive | model =2  two-ratio  Neobatrachia | model =2  two-ratio  Natatanura | model =2  two-ratio  Anura |
| --- | --- | --- | --- | --- | --- | --- |
| *atp6* | -18785.951143 | -18690.62406 | -18690.624045 | **-18769.383790** | **-18747.358988** | -18784.209917 |
| *atp8* |  |  |  |  | -4932.164845 | -4931.463495 |
| *cox1* | -30120.527163 | -29999.208605 | -29999.208605 | **-30097.775598** | **-30087.727544** | -30118.103742 |
| *cox2* | -14748.267499 | -14703.748493 | -14703.748493 | **-14736.508253** | **-14725.244796** | -14747.775627 |
| *cox3* | -16623.951292 | -16512.671503 | -16512.671503 | **-16620.448178** | **-16617.406144** | -16623.021547 |
| *cob* | -26716.375239 | -26502.066074 | -26502.066072 | -26715.747966 | -26714.269941 | -**26705.391176** |
| *nad1* | -22820.427928 | -22718.375747 | -22718.375747 | **-22813.032124** | **-22806.431188** | -22820.300452 |
| *nad2* | -30637.637624 | -30493.381555 | -30493.381555 | **-30633.451375** | **-30623.391651** | -30634.952720 |
| *nad3* | -9036.381146 | -8758.633003 | -8758.633003 | -9036.380193 | -9033.981385 | -9036.288423 |
| *nad4* | -39446.649429 | -39306.541615 | -39306.541615 | **-39442.581094** | **-39435.910720** | -39444.007601 |
| *nad4L* | -7590.966471 | -7569.713478 | -7569.713478 | -7590.027363 | -7590.943906 | -7590.843264 |
| *nad5* | -46120.537591 | -45921.471508 | -45921.471508 | **-46107.651148** | **-46087.959047** | -46120.266545 |
| *nad6* | -12015.164813 | -11810.703959 | -11810.703959 | -12014.463543 | -12014.516092 | -12014.273706 |
| All gene | -285988.440433 |  |  | **-285958.082882** | **-285873.937811** | -285988.239352 |

Bold highlight results that are significantly different to the null model (LRT *p* < 0.05).

LRT = 2Δ (ln L)
