# Supplementary material for: The evolution of mitochondrial genomes in modern frogs (Neobatrachia): nonadaptive evolution of mitochondrial genome reorganization
Source: BMC Genomics. 2014 Aug 20;15(1):691. doi: 10.1186/1471-2164-15-691 (PMC4153901; doi:10.1186/1471-2164-15-691)
Supplement: Supplementary file 6 — Additional file 6: Primers designed for amplifying the complete mitochondrial genome of Glandirana. (DOCX 17 KB) [file 12864_2013_6391_MOESM6_ESM.docx]

Additional file 6

Primers designed for amplifying the complete mitochondrial genome of *Glandirana*

| primer | location | sequence (5’-3’) | product length (bp) |
| --- | --- | --- | --- |
| GIL | 937 | CGTCAGGTCAAGGTGCAG | 1534 |
| GIH | 2471 | CCCGTTTGTCGTGGAGGTT |  |
| GIIL | 2305 | ATCTCCCCGTGAAGAAGCG | 1634 |
| GIIH | 3939 | TCCCTATCAAGGAGGTCCTT |  |
| KIIIL | 3652 | TAAACACCCTAACTACTATTAT | 1514 |
| KIIIH | 5166 | GGTCTGTAYTAGCCTAAGTTTCT |  |
| GIVL | 4926 | ACCCTBGAACTMRTYAAACAA | 1304 |
| GIVH | 6230 | GGCCAAARAATCAGAATARGTG |  |
| GVL | 5924 | CAYGCMGGDCCVTCYGTAGA | 1462 |
| GVH | 7386 | ATTTCRATTTCTTGDGCRTC |  |
| GVIL | 7245 | TTATAGAAGARCTHMTYCACTT | 1555 |
| GVIH | 8800 | GGGCCATAWGTGTTYTCTTG |  |
| KVIIL | 8100 | ATGATCCTGACCATGACAATA | 1894 |
| GVIIH | 9994 | ATCGGTTGTCTTATTTGGACTA |  |
| KVIIIL | 9794 | TCTATGCGGTTCTTCCTTGT | 2729 |
| KVIIIH | 12523 | ATTGAGCGGATTTACTTGCTGCGG |  |
| GXL | 12222 | TCTMCTMGCHATAATTAYCCTT | 1741 |
| GXH | 13963 | GTDGTRTTTGSWTAYTGTGC |  |
| KXIL | 13076 | TCACCCTTATCGCTACAGCCTTCA | 1914 |
| GXIH | 14990 | CTGGWRTAAARTTGTCTGGGTC |  |
| KXIIL | 14744 | CGATTCTTCACATTCCACTTTATCC | 3500 |
| KXIIH | 342 | GGTGTGCTGAGACTTGCATGTGTAA |  |
| KXIIIL | 201 | GCCCTGCACTCTTATAGCTT | 980 |
| KXIIIH | 1181 | ACTTACCGTGTTACGACTTGCC |  |
